# Predicting Brain Age and Gender from Brain Volume Data Using Variational Quantum Circuits

**DOI:** 10.3390/brainsci14040401

**Published:** 2024-04-19

**Authors:** Yeong-Jae Jeon, Shin-Eui Park, Hyeon-Man Baek

**Affiliations:** 1 Department of Health Sciences and Technology, Gachon Advanced Institute for Health Sciences and Technology (GAIHST), Gachon University, Incheon 21999, Republic of Korea; yeong@gachon.ac.kr; 2 Department of BioMedical Science, Lee Gil Ya Cancer and Diabetes Institute, Gachon University, Incheon 21999, Republic of Korea; shineuipark@gmail.com; 3 Department of Molecular Medicine, Lee Gil Ya Cancer and Diabetes Institute, Gachon University, Incheon 21999, Republic of Korea

**Keywords:** brain age prediction, brain age estimation, gender classification, sex classification, structural magnetic resonance imaging, machine learning, quantum machine learning, variational quantum circuit, parameterized quantum circuit, quantum neural network

## Abstract

The morphology of the brain undergoes changes throughout the aging process, and accurately predicting a person’s brain age and gender using brain morphology features can aid in detecting atypical brain patterns. Neuroimaging-based estimation of brain age is commonly used to assess an individual’s brain health relative to a typical aging trajectory, while accurately classifying gender from neuroimaging data offers valuable insights into the inherent neurological differences between males and females. In this study, we aimed to compare the efficacy of classical machine learning models with that of a quantum machine learning method called a variational quantum circuit in estimating brain age and predicting gender based on structural magnetic resonance imaging data. We evaluated six classical machine learning models alongside a quantum machine learning model using both combined and sub-datasets, which included data from both in-house collections and public sources. The total number of participants was 1157, ranging from ages 14 to 89, with a gender distribution of 607 males and 550 females. Performance evaluation was conducted within each dataset using training and testing sets. The variational quantum circuit model generally demonstrated superior performance in estimating brain age and gender classification compared to classical machine learning algorithms when using the combined dataset. Additionally, in benchmark sub-datasets, our approach exhibited better performance compared to previous studies that utilized the same dataset for brain age prediction. Thus, our results suggest that variational quantum algorithms demonstrate comparable effectiveness to classical machine learning algorithms for both brain age and gender prediction, potentially offering reduced error and improved accuracy.

## 1. Introduction

Neuroimaging-derived brain age serves as a valuable biomarker for monitoring the progression of brain-related conditions and aging [1]. This metric, often termed “brain age,” is calculated using machine learning algorithms applied to magnetic resonance imaging (MRI) data to predict an individual’s chronological age. The disparity between the predicted brain age and the actual chronological age reflects deviations from typical age trajectories and is utilized to assess brain health [1]. Elevated brain age relative to chronological age has been correlated with diminished cognitive abilities. Moreover, mental health characteristics, such as Alzheimer’s disease [2], mild cognitive impairment [2], focal epilepsy [3], multiple sclerosis [4], traumatic brain injury [5], schizophrenia [6,7], bipolar disorder [8], major depressive disorder [9], etc., have been associated with an increased brain age difference. These findings underscore the significance of the brain age difference as a biomarker for assessing brain health. The number of publications related to these studies is increasing every year [1].

Gender classification based on neuroimaging data has emerged as a crucial area of research with significant implications across various domains, including neuroscience, medicine, and psychology [10,11,12]. The ability to accurately classify gender from neuroimaging data offers valuable insights into the inherent neurological differences between males and females [10,11,12]. For instance, Flint et al. [10] demonstrated an increased misclassification in transgender women when employing structural MRI data for biological sex classification. Understanding these characteristics is essential for unraveling the complexities of brain structure, function, and development, as well as for addressing gender-related disparities in health and cognition. Moreover, gender classification from neuroimaging data contributes to the elucidation of sex-specific brain disorders and conditions. This capability enables researchers and clinicians to discern gender-specific patterns, thereby facilitating early detection, intervention, and treatment of neurological disorders that may manifest differently between males and females.

Numerous machine learning studies have sought to predict both brain age and gender, primarily utilizing structural MRI data [1,2,3,4,5,6,7,8,9,10,11,12]. Structural MRI scans provide a plethora of brain morphological features, making them a preferred choice for investigating age-related brain changes across various disorders and conditions [1]. The literature encompasses a diverse array of methodological approaches, ranging from classical machine learning methodologies [13,14,15,16] to those employing deep learning techniques [17,18,19,20,21,22,23]. Nevertheless, there seems to be a noticeable lack of research employing quantum machine learning for brain age and gender prediction.

Quantum machine learning has emerged as a promising tool to enhance classical machine learning techniques [24]. Research indicates that both quantum and quantum-inspired computing models have the potential to optimize the training process of conventional models, resulting in improved prediction accuracy for target functions with reduced iteration requirements [25,26]. Several studies have highlighted the practical advantages of quantum machine learning algorithms, demonstrating their superior performance over classical counterparts in predicting complex medical outcomes [25] and image restoration [26]. Among various quantum machine learning methods, parameterized quantum circuits (PQCs), variational quantum circuits (VQCs), or quantum neural networks (QNNs) stand out as particularly promising. For instance, researchers have utilized hybrid quantum neural networks to discover drug molecules [25] and recover contaminated ghost images [26], showcasing superior performance compared to classical counterparts with fewer iterations and higher accuracy, especially when dealing with limited datasets. This suggests their potential for addressing pharmacological and medical challenges, such as predicting patient responses to different medications or evaluating patient prognosis and diagnosis.

This study investigates the application of VQC in predicting brain age and gender using brain morphological features derived from structural MRI data. To the best of our knowledge, these applications represent a novel endeavor. We aim to assess the performance of VQC in comparison with classical machine-learning algorithms. The goal of this study is to explore the potential of quantum machine learning models in predicting brain age and gender based on brain morphometric data, providing invaluable insights into age-related disorders.

## 2. Materials and Methods

### 2.1. Description of Dataset

**Figure 1:**
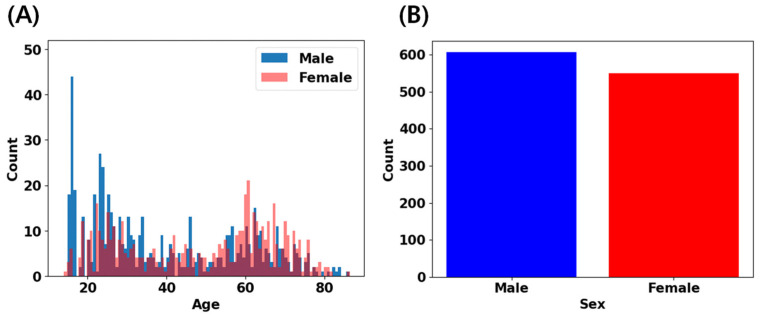
Age and sex distribution across datasets: (**A**) the datasets exhibit bimodal-like age distribution (e.g., young and elderly). (**B**) Sex distribution across the datasets reveals a balanced representation of male and female samples.

In this study, we utilized three primary datasets: the IXI dataset (n = 563, age range 18–88 years, https://brain-development.org [14] (accessed on 27 April 2023)), the CAU dataset (n = 156, age range 55–83 years [15]), and an in-house collected dataset (n = 438, age range 14–89 years [27,28,29]). All participants included in our analysis underwent careful screening following local study protocols to confirm their status as healthy individuals without a history of neurological, psychiatric, or major medical conditions. T1-weighted MRI scans were acquired using either 1.5T or 3T scanners. Detailed information regarding the acquisition protocols for each dataset can be found in the corresponding references [14,15,27,28,29]. Figure 1 and Table 1 provide an overview of the age and gender distributions across our datasets. For each distribution of datasets, the details are provided in Supplementary Figure S1 and Tables S1–S3. Ethical approvals and informed consents were locally obtained for each dataset to ensure compliance with relevant research ethics guidelines.

### 2.2. Image Processing and Feature Extraction

The structural brain T1-weighted MRI scans of all subjects were processed using the FastSurfer v2.1.0 [30], except for the CAU dataset, which had been processed using FreeSurfer [31] run on Ubuntu Linux operating system version 22.04 LTS and was provided in a spreadsheet format, not as raw images. FastSurfer, an alternative version of FreeSurfer, employs deep learning techniques for structural MRI processing. The FastSurfer brain segmentations were carried out on Google Colab using the ‘Tutorial_FastSurferCNN_QuickSeg.ipynb’ notebook. In brief, cortical and subcortical segmentation for each subject was conducted on their T1-weighted image through a series of steps, including skull stripping, segmentation of cortical gray and white matter, and identification of subcortical structures. Further technical details about the pipeline can be found in reference [30]. Notably, this method is highly efficient, taking only a few minutes per subject.

This study utilized estimated subcortical and cortical volume parcellation data. Based on previous studies [14,15], we selected 34 segmentation features from the available 95 labels (refer to Table 2), and later reduced these to 17 features using principal component analysis (PCA) decomposition for age and gender prediction models. This reduction was partly necessitated by the limited qubits available for quantum machine learning algorithms.

### 2.3. Machine Learning Algorithms

Brain age and gender prediction were performed using the scikit-learn library [32] for classical machine learning algorithms and the tensorcircuit package [33] for quantum machine learning algorithms. The tensorcircuit package was selected for its efficiency and ability to utilize a relatively large number of qubits in our experimental environment, allowing us to employ up to 17 qubits in our case. All machine learning algorithms were executed on Google Colab. 

For brain age prediction models, we employed the following six classical machine learning models in scikit-learn: linear regression (LR), support vector regression (SVR) with parameters {‘svr__C’: 15.0, ‘svr__cache_size’: 200, ‘svr_coef0’: 0.0, ‘svr__coef0’: 0.0, ‘svr__degree’: 3, ‘svr__epsilon’: 0.2, ‘svr__gamma’: ‘scale’, ‘svr_kernel’: ‘rbf’, ‘svr__max_iter’: −1, ‘svr__shrinking’: True, ‘svr__tol’: 0.001, ‘svr_verbose’: False}, extreme gradient boosting (XGBoost) with parameters {‘alpha’: 0.9, ‘çcp_alpha’: 0.0, ‘criterion’: ‘friedman_mse’, ‘init’: None, ‘learning_rate’: 0.1, ‘loss’: ‘squared_error’, ‘max_depth’: 3, ‘max_features’: None, ‘max_leaf_nodes’: None, ‘min_impurity_decrease’: 0.0, ‘min_samples_leaf’: 1, ‘min_samples_split’: 2, ‘min_weight_fraction_leaf’: 0.0, ‘n_estimators’: 100, ‘n_iter_no_change’: None, ‘random_state’: None, ‘subsample’: 1.0, ‘tol’: 0.0001, ‘validation_fraction’: 0.1, ‘verbose’: 0, ‘warm_start: False}, random forest (RF) with parameters {‘bootstrap’: True, ‘ccp_alpha’: 0.0, ‘criterian’: ‘squared_error’, ‘max_depth’: None, ‘max_features’: 1.0, ‘max_leaf_nodes’: None, ‘max_samples’: None, ‘min_impurity_decrease’: 0.0, ‘min_samples_leaf’: 1, ‘min_samples_split’: 2, ‘min_weight_fraction_leaf’: 0.0, ‘n_estimators’: 100, ‘n_jobs’: None, ‘oob_score’: False, ‘random_state’: None, ‘verbose’: 0, ‘warm_start’: False}, Bayesian ridge (BR) with parameters {‘alpha_1’: 1 × 10^−6^, ‘alpha_2’: 1 × 10^−6^, ‘alpha_init’: None, ‘compute_score’: False, ‘copy_X’: True, ‘fit_intercept’: True, ‘lambda_1’: 1 × 10^−6^, ‘lambda_2’: 1 × 10^−6^, ‘lambda_init’: None, ‘n_iter’: 300, ‘tol’: 0.001, ‘verbose’: False}, and multi-layer perceptron (MLP) regression with parameters {‘activation’: ‘relu’, ‘alpha’: 0.0001, ‘batch_size’: 10, ‘beta_1’: 0.9, ‘beta_2’: 0.999, ‘early_stopping’: True, ‘epsilon’: 1 × 10^−8^, ‘hidden_layer_sizes’: (17, 17, 17, 17, 17, 17, 17, 17, 17, 17), ‘learning_rate’: ‘constant’, ‘learning_rate_init’: 0.01, ‘max_fun’: 15,000, ‘max_iter’: 1000, ‘momentum’: 0.9, ‘n_iter_no_change’: 10, ‘nesterouvs_momentum’: True, ‘power_t’: 0.5, ‘random_state’: None, ‘shuffle’: True, ‘solver’: ‘adam’, ‘tol’: 0.0001, ‘validation_fraction’: 0.1, ‘verbose’: True, ‘warm_start’: False}. For gender classification, we utilized logistic regression (LR) with parameters {‘C’: 1.0, ‘class_weight’: None, ‘dual’: False, ‘fit_intercept’: True, ‘intercept_scaling’: 1, ‘l1_ratio’: None, ‘max_iter’: 100, ‘multi_class’: ‘auto’, ‘n_jobs’: None, ‘penalty’: ‘l2’, ‘random_state’: None, ‘solver’: ‘lbfgs’, ‘tol’: 0.0001, ‘verbose’: 0, ‘warm_start’: False}, XGBoost, support vector classification (SVC) with parameters {‘C’: 1.0, ‘break_ties’: False, ‘cache_size’: 200, ‘class_weight’: None, ‘coef0’: 0.0, ‘decision_function_shape’: ‘ovr’, ‘degree’: 3, ‘gamma’: ‘scale’, ‘kernel’: ‘rbf’, ‘max_iter’: −1, ‘probability’: False, ‘random_state’: None, ‘shrinking’: True, ‘tol’: 0.001, ‘verbose’: False}, RF with parameters {‘bootstrap’: True, ‘oop_alpha’: 0.0, ‘class_weight’: None, ‘criterion’: ‘gini’, ‘max_depth’: None, ‘max_features’: ‘sqrt’, ‘max_leaf_nodes’: None, ‘max_samples’: None, ‘min_impurity_decrease’: 0.0, ‘min_samples_leaf’: 1, ‘min_samples_split’: 2, ‘min_weight_fraction_leaf’: 0.0, ‘n_estimators’: 100, ‘n_jobs’: None, ‘oob_score’: False, ‘random_state’: None, ‘verbose’: 0, ‘warm_start’: False}, MLP with the same parameters as in brain age estimation, and k-nearest neighbor (KNN) with parameters {‘algorithm’: ‘auto’, ‘leaf_size’: 30, ‘metric’: ‘minkowski’, ‘metric_params’: None, ‘n_jobs’: None, ‘n_neighbors’: 5, ‘p’: 2, ‘weights’: ‘uniform’}. 

**Figure 2:**
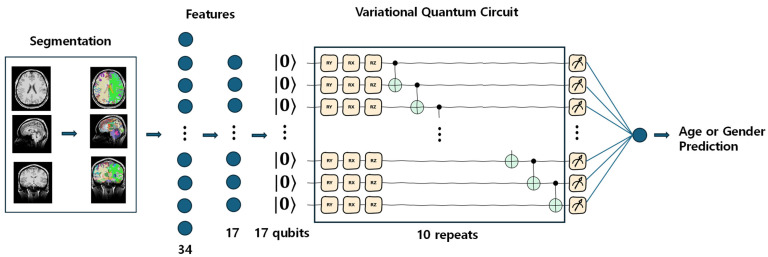
Variational quantum circuit (VQC) architecture for brain age regression and gender classification. T1-weighted structural MRI data undergo segmentation and feature selection, resulting in 34 features. These features are normalized and reduced to 17 elements using principal component analysis (PCA). The 17 features are then fed into the VQC, where trainable operations (Rx, Ry, Rz) and CNOT operations are applied across 10 blocks. After measurements, the outputs are combined into a single layer for brain age prediction or gender classification. Note that the blue arrows represent the direction of forward processing, and the blue circles denote individual features.

In quantum machine learning models, we used variational quantum circuits for age prediction and gender classification tasks. Our VQC model was implemented based on the ‘Quantum Machine Learning for Classification Tasks’ tutorial notebook [33]. We adapted the Ising ZZ coupling gates to CNOT gates (Figure 2). The quantum logic gates used in this study are detailed in Table 3. The quantum circuit in Figure 2 was created using the Pennylane framework [34].

### 2.4. Model Training and Evaluation

Before applying PCA embedding to the model input, we utilized MinMaxScaler from the scikit-learn library to scale the features between zero and one. This normalized feature vector serves as the input for both classical and quantum machine learning algorithms. Focusing on the quantum model, the feature vector underwent transformation into a quantum layer within the VQC. This quantum layer comprised three components: embedding (PCA embedding was employed here), variational layers, and measurement. In our study, we utilized 17 qubits and constructed 10 repeated blocks for the VQC architecture (Figure 2). The normalized classical features were encoded into the quantum Hilbert space, with the resulting quantum state representing the input data from the preceding classical layer. Each variational layer within the VQC consisted of two parts: rotations with trainable parameters and control gates, typically subsequent to CNOT operations (Figure 2 and Table 3). These rotations acted as quantum gates, transforming the encoded input data based on variational parameters, whereas the CNOT operations entangled the qubits in the quantum layer, facilitating the creation of quantum superposition. Each block contained three layers. In the measurement component, all the qubits were measured and summed at a single node. Subsequently, a sigmoid activation function was applied to produce the final output. Thus, the output of the VQC provided predictions of brain age or gender values. The performance of the VQC model was compared with that of classical machine learning models.

For model training, the preprocessed data were shuffled and distributed once into the training (80%) and testing (20%) sets. We selected this split ratio to ensure sufficient data for training the model while preserving a reasonable portion for testing purposes. The split was performed randomly. 

To enhance the representativeness of the dataset and mitigate inadvertent biases, the order of the samples in the training set was shuffled at each epoch, whereas it remained unchanged in the test set. For optimization, we employed an adaptive moment estimation (ADAM) optimizer with a learning rate set to 0.01.

To evaluate the effectiveness of the brain age prediction model, we primarily used the mean absolute error (*MAE*) metric. This metric measures the discrepancy between the predicted brain age ($\hat{y}$) and the corresponding chronological age *(y*) for each sample in our dataset. The MAE is defined as follows:

$$MAE=\frac{1}{N}\sum_{i=1}^{N}{|\hat{y}}_{i}-y_{i}|,$$

where *N* is the number of samples in the dataset. The model’s successful performance is indicated by the low values of the MAE. Other regression metrics, such as the mean squared error (MSE), root mean squared error (RMSE), and r-squared, were also estimated.

On the other hand, to evaluate the performance of the gender classification model, we primarily used the accuracy score defined as follows:

$$Accuracy=\frac{Number\;of\;correct\;predictions}{Total\;number\;of\;predictions}$$

Other classification metrics such as precision, recall, and f1-score were also estimated.

All models were implemented in Python and executed on Google Colab. The classical machine learning algorithms were implemented with scikit-learn, while the quantum machine learning algorithm was implemented with the Tensorcircuit framework. Additionally, we conducted an experiment by training the classical and quantum machine learning models with the same hyperparameters on varying sizes of training data, including 57, 115, 231, 462, 694, and 925 samples. 

## 3. Results

### 3.1. Algorithm Performance for Brain Age Prediction

**Figure 3:**
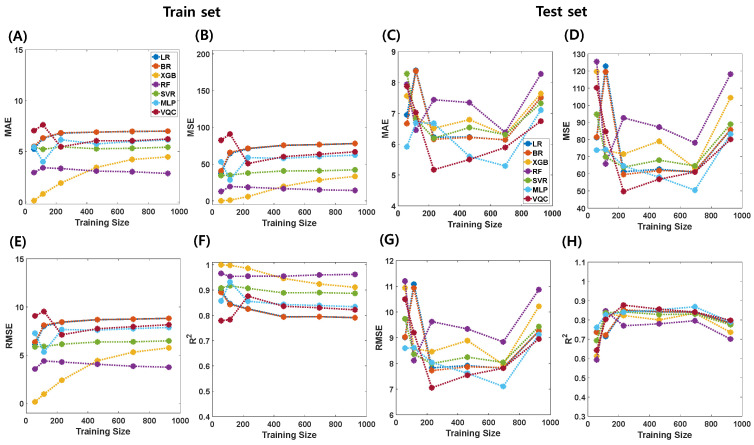
Relationship between training sample sizes and performance of classical (LR, BR, XGB, RF, SVR, MLP) and quantum machine learning (VQC) models for brain age predictions (plots display MAE (**A**,**C**), MSE (**B**,**D**), RMSE (**E**,**G**), and R^2^ (**F**,**H**) values for both train (**A**,**B**,**E**,**F**) and test (**C**,**D**,**G**,**H**) sets against training size).

The performance of each algorithm in the combined dataset is depicted in Figure 3 and Table 4 for both the training set (left four figures) and the hold-out test set (right four figures). Additional metrics, including mean absolute error (MAE), mean squared error (MSE), root mean squared error (RMSE), and r-squared (R^2^), are presented. For a more comprehensive view, performance metrics for various training sample sizes are detailed in Supplementary Tables S4–S8.

The prediction performance varied with the regression algorithms. When the training sample size was 925, the best prediction performance was achieved using VQC (MAE = 6.744, MSE = 80.092, MRSE = 8.949, and R^2^ = 0.798), whereas the worst performance was observed with RF (MAE = 8.275, MSE = 118.161, RMSE = 10.870, and R^2^ = 0.701). Similarly, with 694 samples in the training set, MLP demonstrated the best performance (MAE = 5.293, MSE = 50.448, RMSE = 7.103, and R^2^ = 0.868), whereas RF exhibited the worst performance (MAE = 6.382, MSE = 78.092, MRMSE = 8.837, and R^2^ = 0.796). For the 462 samples in the training set, VQC exhibited the best performance (MAE = 5.502, MSE = 56.829, RMSE = 7.539, R^2^ = 0.856), whereas RF exhibited the worst performance (MAE = 7.348, MSE = 87.258, RMSE = 9.341, R^2^ = 0.780). For 231 samples in the training set, VQC outperformed the other methods (MAE = 5.171, MSE = 49.714, RMSE = 7.051, and R^2^ = 0.877), whereas RF exhibited the worst performance (MAE = 7.439, MSE = 92.641, RMSE = 9.625, and R^2^ = 0.770). Furthermore, with 115 samples, RF achieved the best performance (MAE = 6.452, MSE = 65.773, RMSE = 8.110, and R^2^ = 0.847), whereas LR achieved the worst performance (MAE = 8.391, MSE = 122.784, RMSE = 11.081, and R^2^ = 0.714). Finally, with 57 samples in the training set, MLP exhibited the best performance (MAE = 5.914, MSE = 73.814, RMSE = 8.591, R^2^ = 0.761), whereas SVR and RF demonstrated the worst performances (MAE = 8.283, MSE = 94.746, RMSE = 9.734, R^2^ = 0.693, and MAE = 7.938, MSE = 125.458, RMSE = 11.201, R^2^ = 0.593, respectively). 

### 3.2. Algorithm Performance for Gender Prediction

**Figure 4:**
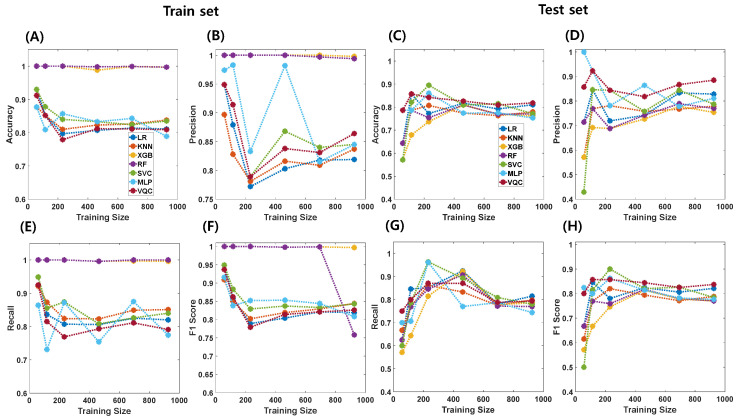
Relationship between training sample sizes and performance of classical (LR, KNN, XGB, RF, SVC, MLP) and quantum machine learning (VQC) models for gender predictions (plots display accuracy (**A**,**C**), precision (**B**,**D**), recall (**E**,**G**), and f1-score (**F**,**H**) for both train (**A**,**B**,**E**,**F**) and test (**C**,**D**,**G**,**H**) sets against training size).

The gender classification performance of each algorithm on the combined dataset is visualized in Figure 4 and summarized in Table 5 for both the training and holdout test sets. The prediction performance varied across classification algorithms. Key metrics such as accuracy, precision, recall, and f1-score values are presented. The detailed results for the different training sample sizes are shown in Supplementary Tables S9–S13.

In gender classification tasks, accuracy, precision, recall, and f1-score values varied across different training sample sizes. For instance, with a training sample size of 925, accuracy ranged from 0.753 to 0.818, with the highest accuracy achieved by VQC and the lowest by XGBoost. Similarly, precision values ranged between 0.754 and 0.885, recall values ranged between 0.744 and 0.815, and f1-score values ranged from 0.770 to 0.837. VQC consistently demonstrated the best prediction performance across various sample sizes, whereas XGBoost exhibited the lowest performance. The performance trends for the other sample sizes followed a similar pattern, with VQC consistently outperforming the other algorithms in terms of accuracy, precision, recall, and F1-score metrics.

### 3.3. Comparative Study for Brain Age Prediction

For comparative analysis, we constructed a VQC model to predict brain age using the IXI and CAU sub-datasets. The model’s performance metrics were as follows: in the IXI dataset, the model achieved an MAE of 6.265, MSE of 65.812, RMSE of 8.106, and R^2^ of 0.759 on the training set (N = 450), and an MAE of 7.201, MSE of 83.074, RMSE of 9.114, and R^2^ of 0.679 on the test set (N = 112) (Table 6).

For the CAU dataset, the model’s performance on the training data (N = 109) resulted in MAE = 3.451, MSE = 17.992, RMSE = 4.242, and R^2^ =0.587, whereas on the test set (N = 47), the performance yielded MAE = 3.302, MSE = 16.675, RMSE = 4.083, and R^2^ =0.425 (Table 7).

## 4. Discussion

In this study, we conducted a comprehensive comparison between quantum machine learning (QML) and classical machine learning (CML) algorithms for brain age regression and gender classification using combined and benchmark datasets. Our findings demonstrate that QML algorithms, particularly variational quantum circuits (VQCs), either outperform or perform comparably to classical algorithms in both tasks.

For brain age prediction, the performance of various algorithms varies significantly, underscoring the importance of algorithm selection. Notably, VQCs consistently exhibit superior performance across different sample sizes, displaying lower mean absolute error (MAE), mean squared error (MSE), and root mean squared error (RMSE) values and higher r-squared (R^2^) scores than the other algorithms. This suggests the potential of QML approaches, particularly VQCs, for accurately predicting brain age based on structural MRI findings. Conversely, random forest (RF) consistently showed comparatively inferior performance, especially in larger sample sizes, highlighting its limitations in handling complex data relationships. Additionally, the performance of linear regression (LR) degraded notably with smaller sample sizes, indicating susceptibility to overfitting or inadequate model complexity. In contrast, the multi-layer perceptron (MLP) demonstrated robust performance across various sample sizes, indicating its adaptability to diverse dataset characteristics.

Similarly, in gender prediction tasks, observed variations in accuracy, precision, recall, and F1-score values across different training sample sizes underscore the significance of both algorithm selection and dataset characteristics. Once again, VQCs consistently outperformed other classical machine learning algorithms across varying sample sizes, achieving superior metrics for all performance measures. This consistent superiority highlights the potential of QML, particularly VQCs, in gender classification tasks, attributed to its ability to capture complex data relationships and generalize across different sample sizes.

Furthermore, the comparative analysis of the VQC model’s performance in predicting brain age using IXI and CAU sub-datasets provided valuable insights into its effectiveness across diverse datasets. Our findings indicate that VQC outperforms previous studies [14,15] that utilized Automatic Relevance Determination (Table 6) and Bayesian Ridge (Table 7) algorithms, achieving better brain age prediction metrics using brain morphometric data. These results suggest the superiority of VQC in accurately predicting brain age across different datasets.

One interesting finding is that, although VQC did not demonstrate superior performance compared to other algorithms in the training set for both brain age prediction and gender classification tasks, it exhibited excellent performance in the test set (Figure 3 and Figure 4). This result implies that QML may possess better generalization capabilities than CML algorithms. The quantum advantage might indeed have played a role in enabling this enhanced performance [35].

Overall, our study contributes to the expanding body of literature on QML applications in healthcare and neuroscience. While our findings demonstrate promising results for VQC in brain age regression and gender classification tasks, further research is warranted to explore its generalizability and integration into clinical practice for neurological research. 

The limitation of this study is that, first, we generally could not demonstrate that our model outperforms deep-learning-based models in other previous studies [17,18,19,20,21,22,23]. For instance, in the brain age prediction task, Wang et al. [36] examined a T1-weighted MRI dataset of 3688 dementia-free participants with a mean age of 66 years, utilizing a convolutional neural network (CNN) deep learning algorithm to predict brain age. They achieved a mean absolute error (MAE) of 4.45 years. Hwang et al. [18] explored the feasibility and clinical relevance of brain age prediction using axial T2-weighted images of healthy subjects with a deep CNN model. The CNN model was trained with 1530 scans, and the MAE evaluated the performance between the predicted age and the chronological age based on an internal and external test dataset. The model showed MAEs of 4.22 years in the internal test set and 9.96 years in the external test set. Mendes, S.L et al. [11] employed two public datasets, ABIDE-II and ADHD-200, comprising healthy controls (HC, N = 894), autism spectrum disorder (ASD, N = 251), and attention deficit hyperactivity disorder (ADHD, N = 357) individuals, for age prediction and gender classification tasks. They utilized T1-weighted sMRI scans and preprocessed gray and white matter images using Voxel-Based Morphometry (VBM), and subsequently trained models with 3D convolutional neural networks (CNNs). Their best-performing model, trained on the ADHD-200 dataset, achieved an MAE of 1.43 years and an R^2^ score of 0.62 for age prediction on the test set. For gender classification, the model achieved an AUC-ROC of 0.85, with precision, recall, and F1-score values of 0.84, 0.81, and 0.83, respectively. Conversely, when using the ABIDE-II dataset, the age prediction model yielded an MAE of 1.63 and an R^2^ score of 0.54, while the gender classification model achieved an AUC-ROC of 0.82, with precision, recall, and F1-score values of 0.87, 0.80, and 0.83, respectively. 

As our study did not employ the same datasets as those mentioned above, a direct comparison might be challenging. However, it appears that the deep-learning-based studies cited above demonstrated higher performance metrics than ours, likely owing to commonalities in their methodologies. Specifically, many of these studies minimized or completely avoided the preprocessing steps, trained deep learning models directly on raw images, or used minimal transformations. In contrast, our study involved preprocessing to extract brain morphometry features, and the limited number of qubits required for VQCs hindered us from training the model using all the features, potentially leading to information loss. To address these challenges, hybrid approaches that combine classical and quantum machine learning [25,26] and employ techniques such as quanvolutional neural networks [37,38] or data reuploading [39] could potentially yield better results. In addition, our study did not demonstrate the clinical utility of age prediction and gender classification, which may require disease-specific or atypical data. Therefore, future research should focus on applying improved models to a broader range of applications, including clinical scenarios, to demonstrate their practical relevance. 

## 5. Conclusions

In conclusion, our study compared quantum and classical machine learning algorithms for brain age regression and gender classification. We found that variational quantum circuits (VQCs) consistently outperformed or were comparable to classical algorithms across both tasks. Although VQCs consistently showed superior performance, limitations such as information loss due to preprocessing and qubit constraints were noted. Future research should explore hybrid approaches or advanced techniques to address these challenges and demonstrate their practical relevance in clinical scenarios.

## Figures and Tables

**Table 1:** Demographics of subjects included in this study.

Age Range	No. of Subjects		
	Male	Female	Total
14–19	96	27	123
20–29	159	120	279
30–39	86	54	140
40–49	59	60	119
50–59	69	83	152
60–69	95	134	229
70–79	36	66	102
80–89	7	6	13
Total	607	550	1157

**Table 2:** The 34 selected features from MRI brain volume segmentation data.

No.	Feature	No.	Feature
1	Left white matter	18	Right white matter
2	Left lateral ventricle	19	Right lateral ventricle
3	Left inferior lateral ventricle	20	Right inferior lateral ventricle
4	Left cerebellum white matter	21	Right cerebellum white matter
5	Left cerebellum cortex	22	Right cerebellum cortex
6	Left thalamus proper	23	Right thalamus proper
7	Left caudate	24	Right caudate
8	Left putamen	25	Right putamen
9	Left pallidum	26	Right pallidum
10	Left hippocampus	27	Right hippocampus
11	Left amygdala	28	Right amygdala
12	Left accumbens area	29	Right accumbens area
13	Left ventralDC	30	Right ventralDC
14	Left choroid plexus	31	Right choroid plexus
15	Left cerebral cortex	32	Right cerebral cortex
16	Cerebrospinal fluid	33	Brain stem
17	Third ventricle	34	Fourth ventricle

**Table 3:** Quantum logic gates used in this study.

Name	Purpose	Matrix	Symbol
Parameterized X Rotation	Rotates the qubit by θ around the x-axis	$\left[\begin{array}{cc} cos(\frac{\theta }{2}) & -isin(\frac{\theta }{2})\\ -isin(\frac{\theta }{2}) & cos(\frac{\theta }{2}) \end{array}\right]$	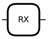
Parameterized Y Rotation	Rotates the qubit by θ around the y-axis	$\left[\begin{array}{cc} cos(\frac{\theta }{2}) & -sin(\frac{\theta }{2})\\ sin(\frac{\theta }{2}) & cos(\frac{\theta }{2}) \end{array}\right]$	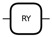
Parameterized Z Rotation	Rotates the qubit by θ around the z-axis	$\left[\begin{array}{cc} exp(-\frac{i\theta }{2}) & 0\\ 0 & exp(\frac{i\theta }{2}) \end{array}\right]$	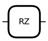
Controlled NOT (CNOT)	Entangle two qubits in a quantum circuit	$\left[\begin{array}{cc} \begin{array}{cc} 1 & 0\\ 0 & 1 \end{array} & \begin{array}{cc} 0 & 0\\ 0 & 0 \end{array}\\ \begin{array}{cc} 0 & 0\\ 0 & 0 \end{array} & \begin{array}{cc} 0 & 1\\ 1 & 0 \end{array} \end{array}\right]$	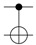

**Table 4:** Age prediction performance of various machine learning regressors.

Regressors	Train (N = 925)				Test (N = 231)			
	MAE	MSE	RMSE	R^2^	MAE	MSE	RMSE	R^2^
LR	6.978	77.987	8.831	0.791	7.506	85.695	9.257	0.784
BR	6.982	78.013	8.833	0.791	7.512	85.733	9.259	0.783
XGBoost	4.437	33.259	5.767	0.911	7.639	104.394	10.217	0.736
RF	2.809	14.118	3.757	0.962	8.275	118.161	10.870	0.701
SVR	5.395	42.177	6.494	0.887	7.324	88.986	9.433	0.775
MLP	6.118	62.193	7.886	0.834	7.103	83.184	9.121	0.790
VQC	6.200	66.674	8.165	0.822	6.744	80.092	8.949	0.798

LR: linear regression; BR: Bayesian ridge; XGBoost: extreme gradient boosting; RF: random forest; SVR: support vector regression; MLP: multilayer perceptron; VQC: variational quantum circuit.

**Table 5:** Gender prediction performance of various machine learning classifiers.

Classifiers	Train (N = 925)				Test (N = 231)			
	Accuracy	Precision	Recall	F1-Score	Accuracy	Precision	Recall	F1-Score
LR	0.811	0.819	0.820	0.819	0.810	0.828	0.815	0.821
KNN	0.838	0.837	0.851	0.844	0.779	0.779	0.798	0.788
XGBoost	0.997	0.998	0.996	0.997	0.762	0.754	0.786	0.770
RF	0.997	0.994	1.000	0.758	0.770	0.770	0.770	0.770
SVC	0.835	0.845	0.840	0.843	0.771	0.787	0.780	0.784
MLP	0.789	0.845	0.774	0.808	0.753	0.811	0.744	0.776
VQC	0.809	0.864	0.791	0.826	0.818	0.885	0.794	0.837

LR, logistic regression; KNN, k-nearest neighbor; XGBoost, extreme gradient boosting; RF, random forest; SVC, support vector classifier; MLP, multilayer perceptron; VQC, variational quantum circuit.

**Table 6:** Comparative study of IXI dataset for brain age prediction in the training data (N = 450) and prediction performance (N = 113).

Author	Method	Model Performance (MAE)	Prediction Performance (MAE)
Han, J. et al. [14]	ARD	7.4790	8.0453
Proposed	VQC	6.265	7.201

ARD: Automatic Relevance Determination, VQC: variational quantum circuit; MAE: mean squared error.

**Table 7:** Comparative study of CAU dataset for brain age prediction in the training data (N = 109) and prediction performance (N = 47).

Author	Method	MAE	MSE	RMSE	R^2^
Simfukwe, C. et al. [15]	BR	3.310	18.280	4.280	0.300
Proposed	VQC	3.302	16.675	4.083	0.425

BR, Bayesian ridge; VQC, variational quantum circuit; MAE, mean absolute error; MSE, mean squared error; RMSE, root mean squared error.

## Data Availability

Partial data used in this study are publicly available and be accessed directly from the Information eXtraction from Images (https://brain-development.org (accessed on 27 April 2023)) websites and (Algorithms—Google Drive).

## Supplementary Materials

The following supporting information can be downloaded from: https://www.mdpi.com/article/10.3390/brainsci14040401/s1, Table S1: Demographics of in-house collected dataset, Table S2: Demographics of IXI dataset, Table S3: Demographics of CAU dataset, Table S4: Age prediction performance of various machine learning regressors (training data, N = 694, hold-out test data, N = 173), Table S5: Age prediction performance of various machine learning regressors (training size, N = 462, hold-out test data, N = 115), Table S6: Age prediction performance of various machine learning regressors (training size, N = 231, hold-out test data, N = 57), Table S7: Age prediction performance of various machine learning regressors (training size, N = 115, hold-out test data, N = 28), Table S8: Age prediction performance of various machine learning regressors (training size, N = 57, hold-out test data, N = 14), Table S9: Gender prediction performance of various machine learning classifiers (training data, N = 694, hold-out test data, N = 173), Table S10: Gender prediction performance of various machine learning classifiers (training data, N = 462, hold-out test data, N = 115), Table S11: Gender prediction performance of various machine learning classifiers (training data, N = 231, hold-out test data, N = 57), Table S12: Gender prediction performance of various machine learning classifiers (training data, N = 115, hold-out test data, N = 28), Table S13: Gender prediction performance of various machine learning classifiers (training data, N = 57, hold-out test data, N = 14). Figure S1: Age and sex distributions across each dataset. (A) in-house collected dataset. (B) IXI dataset. (C) CAU dataset. Figure S2: The scree plot (A) and the cumulative explained variance ratio (B) by the number of principal components.
