# Supplementary material for: Predicting Brain Age and Gender from Brain Volume Data Using Variational Quantum Circuits
Source: Brain Sci. 2024 Apr 19;14(4):401. doi: 10.3390/brainsci14040401 (PMC11048383; doi:10.3390/brainsci14040401)
Supplement: Supplementary file 1 [file brainsci-14-00401-s001.zip › brainsci-2958321-supplementary.pdf]

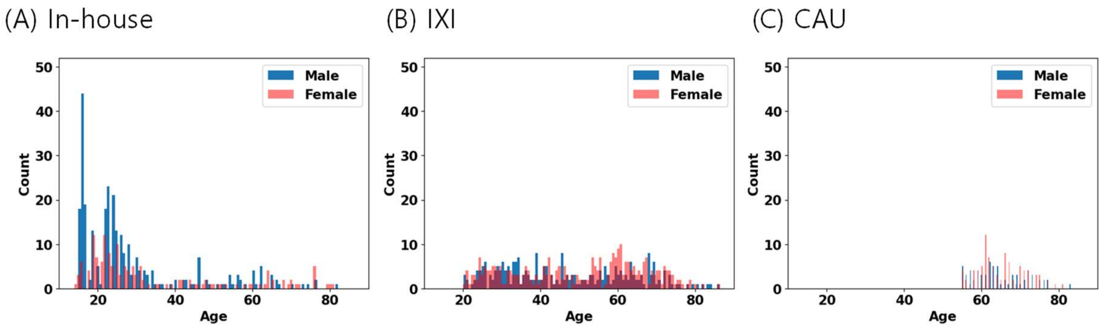

Figure S1. Age and sex distributions across each dataset. (A) in-house collected dataset. (B) IXI dataset. (C) CAU dataset.

Table S1. Demographics of in-house collected dataset.

Age range	No. of subjects		
	Male	Female	Total
14-19	96	26	122
20-29	114	65	179
30-39	26	15	41
40-49	18	12	30
50-59	13	7	20
60-69	16	12	28
70-79	5	10	15
80-89	1	2	3
Total	289	149	438

Table S2. Demographics of IXI dataset.

Age range	No. of subjects		
	Male	Female	Total
14-19	0	1	1
20-29	45	55	100
30-39	60	39	99
40-49	41	48	89
50-59	38	61	99
60-69	47	71	118
70-79	15	34	49
80-89	5	3	8
Total	251	312	563

Table S3. Demographics of CAU dataset.

Age range	No. of subjects		
	**Male**	**Female**	**Total**
14-19	0	0	0
20-29	0	0	0
30-39	0	0	0
40-49	0	0	0
50-59	18	15	33
60-69	32	51	83
70-79	16	22	38
80-89	1	1	2
Total	67	89	156

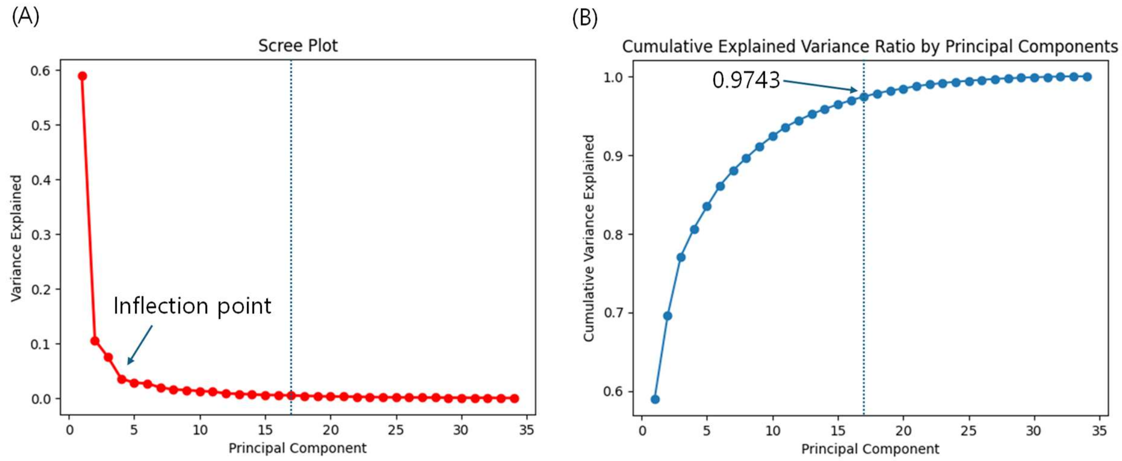

Figure S2. The scree plot (A) and the cumulative explained variance ratio (B) by the number of principal components.

Table S4. Age prediction performance of various machine learning regressors.

**Regressors**	**Train (N=694)**				**Test (N=173)**			
	**MAE**	**MSE**	**RMSE**	**R^2**	**MAE**	**MSE**	**RMSE**	**R^2**
LR	6.941	76.521	8.748	0.795	6.137	61.021	7.812	0.841
BR	6.951	76.566	8.750	0.795	6.143	61.425	7.837	0.840
XGBoost	4.188	28.302	5.320	0.924	6.305	63.321	7.957	0.835
RF	2.974	14.917	3.862	0.960	6.382	78.092	8.837	0.796
SVR	5.288	40.909	6.396	0.890	6.307	64.628	8.039	0.831
MLP	5.958	60.272	7.763	0.839	5.293	50.448	7.103	0.868
VQC	6.029	63.401	7.963	0.830	5.891	61.019	7.811	0.841

LR: linear regression, BR: Bayesian ridge, XGBoost: eXtreme gradient boosting, RF: randomforest, SVR: support vector regression, MLP: multi-layer perceptron, VQC: variational quantum circuit.

Table S5. Age prediction performance of various machine learning regressors.

Regressors	Train (N=462)				Test (N=115)			
	**MAE**	**MSE**	**RMSE**	**R^2**	**MAE**	**MSE**	**RMSE**	**R^2**
LR	6.884	75.517	8.690	0.795	6.229	62.633	7.914	0.842
BR	6.884	75.622	8.696	0.794	6.217	61.887	7.867	0.844
XGBoost	3.403	19.670	4.435	0.946	6.793	78.974	8.887	0.800
RF	3.038	16.506	4.063	0.955	7.348	87.258	9.341	0.780
SVR	5.243	40.672	6.377	0.889	6.539	67.969	8.244	0.828
MLP	5.717	57.861	7.607	0.843	5.600	58.000	7.616	0.853
VQC	6.029	60.127	7.754	0.836	5.502	56.829	7.539	0.856

LR: linear regression, BR: Bayesian ridge, XGBoost: eXtreme gradient boosting, RF: randomforest, SVR: support vector regression, MLP: multi-layer perceptron, VQC: variational quantum circuit.

Table S6. Age prediction performance of various machine learning regressors.

Regressors	Train (N=231)				Test (N=57)			
	**MAE**	**MSE**	**RMSE**	**R^2**	**MAE**	**MSE**	**RMSE**	**R^2**
LR	6.773	71.010	8.427	0.826	6.228	61.251	7.826	0.848
BR	6.809	71.372	8.448	0.825	6.158	59.592	7.720	0.852
XGBoost	1.863	5.781	2.404	0.986	6.513	71.529	8.457	0.823
RF	3.294	18.472	4.298	0.955	7.439	92.641	9.625	0.770
SVR	5.427	37.784	6.147	0.907	6.192	63.877	7.992	0.842
MLP	6.132	58.818	7.669	0.856	6.676	64.565	8.035	0.840
VQC	5.439	50.765	7.125	0.876	5.171	49.714	7.051	0.877

LR: linear regression, BR: Bayesian ridge, XGBoost: eXtreme gradient boosting, RF: randomforest, SVR: support vector regression, MLP: multi-layer perceptron, VQC: variational quantum circuit.

Table S7. Age prediction performance of various machine learning regressors.

Regressors	Train (N=115)				Test (N=28)			
	**MAE**	**MSE**	**RMSE**	**R^2**	**MAE**	**MSE**	**RMSE**	**R^2**
LR	6.268	64.375	8.023	0.846	8.391	122.784	11.081	0.714
BR	6.306	65.874	8.116	0.843	8.375	119.549	10.934	0.721
XGBoost	0.794	0.926	0.962	0.998	6.890	73.675	8.583	0.828
RF	3.364	19.405	4.405	0.954	6.452	65.773	8.110	0.847
SVR	5.189	34.957	5.912	0.917	6.822	69.910	8.361	0.837
MLP	3.951	28.450	5.334	0.932	6.692	74.150	8.611	0.827
VQC	7.599	90.988	9.539	0.783	7.024	84.606	9.198	0.803

LR: linear regression, BR: Bayesian ridge, XGBoost: eXtreme gradient boosting, RF: randomforest, SVR: support vector regression, MLP: multi-layer perceptron, VQC: variational quantum circuit.

Table S8. Age prediction performance of various machine learning regressors.

Regressors	Train (N=57)				Test (N=14)			
	MAE	MSE	RMSE	R^2	MAE	MSE	RMSE	R^2
LR	5.201	37.476	6.122	0.899	6.948	81.119	9.007	0.737
BR	5.481	40.620	6.373	0.891	6.665	81.408	9.023	0.736
XGBoost	0.127	0.026	0.160	1.000	7.564	119.670	10.939	0.612
RF	2.896	12.777	3.574	0.966	7.938	125.458	11.201	0.593
SVR	5.419	34.399	5.865	0.908	8.283	94.746	9.734	0.693
MLP	5.411	53.057	7.284	0.857	5.914	73.814	8.591	0.761
VQC	7.026	82.466	9.081	0.779	7.875	110.248	10.500	0.643

LR: linear regression, BR: Bayesian ridge, XGBoost: eXtreme gradient boosting, RF: randomforest, SVR: support vector regression, MLP: multi-layer perceptron, VQC: variational quantum circuit.

Table S9. Gender prediction performance of various machine learning classifiers.

Classifiers	Train (N=694)				Test (N=173)			
	Accuracy	Precision	Recall	F1-score	Accuracy	Precision	Recall	F1-score
LR	0.814	0.818	0.825	0.821	0.792	0.833	0.781	0.806
KNN	0.826	0.809	0.849	0.829	0.763	0.767	0.775	0.771
XGBoost	0.999	1.000	0.997	0.999	0.769	0.778	0.778	0.778
RF	0.999	0.997	1.000	0.999	0.769	0.789	0.772	0.780
SVC	0.826	0.840	0.826	0.833	0.815	0.844	0.809	0.826
MLP	0.843	0.815	0.875	0.844	0.775	0.778	0.787	0.782
VQC	0.811	0.831	0.811	0.821	0.809	0.867	0.788	0.825

LR: losgistic regression, KNN: k-nearest neighbor, XGBoost: eXtream gradient boosting, RF: randomforest, SVC: support vector classifier, MLP: multi-layer perceptron, VQC: variational quantum circuit.

Table S10. Gender prediction performance of various machine learning classifiers.

Classifiers	Train (N=462)				Test (N=115)			
	Accuracy	Precision	Recall	F1-score	Accuracy	Precision	Recall	F1-score
LR	0.807	0.803	0.806	0.804	0.817	0.742	0.925	0.824
KNN	0.823	0.816	0.823	0.816	0.774	0.758	0.833	0.794
XGBoost	0.988	1.000	0.996	0.998	0.809	0.727	0.923	0.814
RF	0.998	1.000	0.996	0.998	0.809	0.742	0.907	0.817
SVC	0.833	0.868	0.808	0.837	0.809	0.758	0.893	0.820
MLP	0.833	0.982	0.754	0.853	0.774	0.864	0.770	0.814
VQC	0.812	0.838	0.793	0.814	0.826	0.818	0.871	0.844

LR: losgistic regression, KNN: k-nearest neighbor, XGBoost: extreamm gradient boosting, RF: randomforest, SVC: support vector classifier, MLP: multi-layer perceptron, VQC: variational quantum circuit.

Table S11. Gender prediction performance of various machine learning classifiers.

Classifiers	Train (N=231)				Test (N=57)			
	Accuracy	Precision	Recall	F1-score	Accuracy	Precision	Recall	F1-score
LR	0.797	0.772	0.907	0.789	0.772	0.719	0.852	0.780
KNN	0.810	0.781	0.824	0.802	0.807	0.781	0.862	0.820
XGBoost	1.000	1.000	1.000	1.000	0.737	0.688	0.815	0.746
RF	1.000	1.000	1.000	1.000	0.754	0.688	0.846	0.759
SVC	0.840	0.789	0.874	0.829	0.895	0.844	0.964	0.900
MLP	0.857	0.833	0.872	0.852	0.860	0.781	0.962	0.862
VQC	0.779	0.789	0.769	0.779	0.842	0.844	0.871	0.857

LR: losgistic regression, KNN: k-nearest neighbor, XGBoost: extreamm gradient boosting, RF: randomforest, SVC: support vector classifier, MLP: multi-layer perceptron, VQC: variational quantum circuit.

Table S12. Gender prediction performance of various machine learning classifiers.

Classifiers	Train (N=115)				Test (N=28)			
	Accuracy	Precision	Recall	F1-score	Accuracy	Precision	Recall	F1-score
LR	0.852	0.879	0.836	0.857	0.857	0.846	0.846	0.846
KNN	0.852	0.828	0.873	0.850	0.786	0.769	0.769	0.769
XGBoost	1.000	1.000	1.000	1.000	0.679	0.692	0.643	0.667
RF	1.000	1.000	1.000	1.000	0.786	0.769	0.769	0.769
SVC	0.878	0.914	0.855	0.883	0.821	0.846	0.786	0.815
MLP	0.809	0.983	0.731	0.838	0.786	0.923	0.706	0.800
VQC	0.852	0.914	0.815	0.862	0.857	0.923	0.800	0.857

LR: losgistic regression, KNN: k-nearest neighbor, XGBoost: extreamm gradient boosting, RF: randomforest, SVC: support vector classifier, MLP: multi-layer perceptron, VQC: variational quantum circuit.

Table S13. Gender prediction performance of various machine learning classifiers.

Classifiers	Train (N=57)				Test (N=14)			
	Accuracy	Precision	Recall	F1-score	Accuracy	Precision	Recall	F1-score
LR	0.930	0.949	0.949	0.949	0.643	0.714	0.625	0.667
KNN	0.877	0.897	0.921	0.909	0.643	0.571	0.667	0.615
XGBoost	1.000	1.000	1.000	1.000	0.571	0.571	0.571	0.571
RF	1.000	1.000	1.000	1.000	0.643	0.714	0.625	0.667
SVC	0.930	0.949	0.949	0.949	0.571	0.429	0.600	0.500
MLP	0.877	0.974	0.864	0.916	0.786	1.000	0.700	0.824
VQC	0.912	0.949	0.925	0.937	0.786	0.857	0.750	0.800

LR: losgistic regression, KNN: k-nearest neighbor, XGBoost: extreamm gradient boosting, RF: randomforest, SVC: support vector classifier, MLP: multi-layer perceptron, VQC: variational quantum circuit.
